# The Impact of Adverse Childhood Experiences on Therapy Outcome in Adolescents Engaging in Nonsuicidal Self-Injury

**DOI:** 10.3389/fpsyt.2020.505661

**Published:** 2020-11-04

**Authors:** Alexandra Edinger, Gloria Fischer-Waldschmidt, Peter Parzer, Romuald Brunner, Franz Resch, Michael Kaess

**Affiliations:** ^1^Section for Translational Psychobiology in Child and Adolescent Psychiatry, Clinic of Child and Adolescent Psychiatry, Center for Psychosocial Medicine, University Hospital Heidelberg, Heidelberg, Germany; ^2^Clinic of Child and Adolescent Psychiatry, Center for Psychosocial Medicine, University Hospital Heidelberg, Heidelberg, Germany; ^3^Clinic and Policlinic of Child and Adolescent Psychiatry, Psychosomatics and Psychotherapy, University of Regensburg, Regensburg District Hospital, Regensburg, Germany; ^4^University Hospital of Child and Adolescent Psychiatry and Psychotherapy, University of Bern, Bern, Switzerland

**Keywords:** nonsuicidal self-injury, adverse childhood experiences, treatment outcome, adolescents, psychotherapy

## Abstract

**Objective:** Nonsuicidal self-injury (NSSI) is a prevalent and clinically significant behavior. There is a substantial association between adverse childhood experiences (ACEs) and NSSI. However, there are no studies investigating the impact of ACEs on NSSI treatment (psychotherapy) outcome. The aim of this secondary analysis of a randomized controlled trial (RCT) on psychotherapy of NSSI was to investigate the relationship between ACEs and treatment outcome in adolescents engaging in NSSI.

**Method:** A sample of 74 adolescent outpatients engaging in repetitive NSSI (incidents on ≥ 5 days within the last 6 months) was recruited for a RCT. ACEs were assessed by the Childhood Experience of Care and Abuse (CECA) interview before treatment onset. Based on the CECA, participants were divided in two groups: with a history of ACEs (*n* = 30) and without a history of ACEs (*n* = 44). Frequencies of NSSI, depression, and suicide attempts as well as quality of life were measured at three points in time: before treatment onset (baseline; T0), 4 (T1), and 10 months (T2) after treatment onset.

**Results:** Both participants with and without ACEs were able to reduce the frequency of NSSI significantly [χ^2^_(1)_ = 26.72; *p* < 0.001]. Surprisingly, participants with ACEs reached a significantly greater reduction in NSSI frequency within the past 6 months compared to participants without ACEs [χ^2^_(1)_ = 5.08; *p* = 0.024]. There were also substantial and similar improvements regarding depressive symptoms, suicide attempts and quality of life in both groups.

**Conclusion:** ACEs seem to positively predict treatment response in psychotherapy for adolescent NSSI. This is contrary to prior research suggesting ACE as an unfavorable prognostic factor in the treatment of mental disorders.

**Clinical Trial Registration:** Short term therapy in adolescents with self-destructive and risk-taking behaviors; http://www.drks.de; DRKS00003605.

## Introduction

Nonsuicidal self-injury (NSSI) is defined “as the deliberate, self-inflicted damage of body tissue without suicidal intent and for purposes not socially or culturally sanctioned” (International Society for the Study of Self-Injury, ISSS). NSSI is categorized as an independent disorder in need of further study in the fifth edition of the Diagnostic and Statistical Manual of Mental Disorders (DSM-5) (1). It is a highly recurrent behavior and peaks in adolescence (2, 3). Approximately 17–18% of adolescents worldwide are affected (4, 5). The prevalence rate for repetitive NSSI using the criteria of the DSM-5 ranges between 1.5 and 6.7% in a recent community study (6). In clinical samples, NSSI is exhibited by 50–60% of adolescents (7). Although NSSI is associated with a variety of psychiatric disorders, including posttraumatic stress disorder (PTSD) (8) and borderline personality disorder (BPD), it also occurs without any comorbid diagnoses (9).

Nock (10) presented an etiology model to explain the development and maintenance of NSSI. Within the model, he postulates both distal risk factors like specific genetic predispositions for high cognitive and emotional reactivity as well as environmental factors such as childhood maltreatment and hostility/criticism within the familial context. These factors are suggested to result in poor emotion regulation and communication skills, which in turn increase the risk for NSSI (10). The postulated distal risk factors of childhood maltreatment and familial hostility can be summarized under the term “adverse childhood experiences” (ACEs). ACEs refer to distressing and/or traumatic events that occur during childhood, such as abuse, deprivation, and neglect (11). A systematic review consisting of 20 cross-sectional studies found a positive association between childhood maltreatment and NSSI (12). More broadly, ACEs have consistently been identified as significant predictors of NSSI among adolescents from the community (13–15). However, ACEs were also specifically predictive of NSSI within child and adolescent patient samples (7, 16–18).

Concerning different types of ACEs, experiences of neglectful or harsh parenting seem to play a most prominent role. Previous studies revealed highest associations for maternal antipathy and neglect (7). In line with these findings, a strong association of increased parental critique and apathy has been shown (19). However, it is important to note that longitudinal studies revealed reciprocal effects between NSSI and parenting, e.g., a significant impact of NSSI on parents' well-being and therefore on their ability to support their children (20, 21).

Another study found that only child emotional abuse remained significantly associated with NSSI, when different types of ACEs were analyzed simultaneously (18). Also, Brown et al. (22) found that especially emotional neglect and abuse seem to be important in the etiology of NSSI. A recent meta-analysis showed that childhood maltreatment, but in particular emotional abuse, was associated with NSSI (23). Nonetheless, and besides the importance of those experiences above, sexual abuse has been repeatedly shown to be associated with the development and onset of NSSI (7, 24–26).

Not all adolescents with NSSI report a history of ACEs. Previous studies revealed frequencies of 64% among samples of adolescent inpatients engaging in NSSI (7). Within community samples, 53.3% of adolescents with NSSI reported ACEs, most frequently emotional abuse (27). Interestingly, the presence of ACEs was significantly related to automatic functions of NSSI (e.g., affect regulation, anti-dissociative function, or self-punishment) within a study on adolescent inpatients with repetitive NSSI (7). In line with these findings, it was shown that adolescents with greater ACEs showed poorer self-regulation than adolescents without ACEs (28).

There are treatment options which are useful in the treatment of NSSI, like dialectical behavior therapy for adolescents [DBT-A (29, 30)] and mentalization-based treatment for adolescents [MBT-A (31)]. Recently, our working group evaluated a specific short-term program for adolescent NSSI, which shows to be as effective as treatment as usual in reducing NSSI as well as common comorbid symptomatology (32). However, in terms of a personalized medicine (33), no criteria exist—beyond the presence of NSSI—that may guide adequate decision making regarding which treatment is best for the individual patient. Considering the transdiagnostic character of NSSI (34), more specific indicators are needed to provide individuals with the best-fitting therapy to increase effectiveness. Therefore, studies investigating predictors of treatment outcome are warranted in order to facilitate personalized treatment in the future.

Literature postulates that ACEs have a negative impact on treatment outcome. One idea is that the presence of ACEs leads to more severe psychopathology, which in turn causes poorer prognosis concerning therapy outcomes. A study on depressed outpatients found that those with ACEs showed poorer therapy outcomes: patients with ACEs had a longer time to remission, and they needed a combination treatment of antidepressants and psychotherapy significantly more often compared to their counterparts without ACEs (35). Another idea is that ACEs cause attachment problems, which interfere with the therapeutic alliance (36). The therapeutic alliance is one of the common, unspecific curative factors in psychotherapy (37). Thus, this relationship could explain the poor therapy outcomes in patients with ACEs. Another finding is that ACEs cause severe comorbid psychopathology, which occurs at a later point in time (36). Therefore, there might be unfavorable therapy outcomes because of upcoming psychopathology.

In general, higher numbers of negative life events are associated not only with the onset of psychopathology but also with poorer outcomes and greater chances of relapse (38). A meta-analysis of 16 epidemiological studies suggested that ACEs were associated with an elevated risk of developing persistent and recurrent depressive episodes (39). A meta-analysis of 10 clinical trials revealed that ACEs were associated with lack of response or remission during treatment for depression. It was concluded that ACEs predict an unfavorable course of illness and treatment outcome in depression (39). A study with adult dysthymia patients showed similar results: at a 5-year follow-up, patients with experiences of sexual abuse and poor childhood maternal and paternal relationships showed a lower rate of recovery from dysthymic disorder and higher levels of depression compared to participants without ACEs (40). In a study investigating therapy response in substance use disorders, emotional abuse as well as witnessed assaults were negatively related to treatment outcome, whereas physical and sexual abuse were not predictive (41). Another study investigated predictors of therapy outcome in adult outpatient borderline personality disorder (BPD) patients (42). Childhood physical abuse was one of the significant factors that predicted dropout from treatment. Depressive disorders, BPD, and substance use disorders are often comorbid to NSSI, giving a hint that the same might be true for treatment outcomes in NSSI. However, there are no studies to date examining the impact of ACEs on treatment outcome in adolescents engaging in NSSI.

This secondary data analysis of a previously published (32) randomized controlled trial (RCT) on psychotherapy of adolescent NSSI aimed to investigate the impact of ACEs on treatment outcome, which was defined as a reduction in the frequency of NSSI, suicide attempts, a reduction of depressive symptoms, and an increase in quality of life over time. As primary hypothesis, we assumed that adolescents with ACEs would show poorer treatment outcomes regarding NSSI (reduction of NSSI frequencies within the past 6 months) compared to adolescents with no history of ACEs. As secondary hypothesis, we assumed that adolescents with ACEs would show poorer treatment outcomes regarding suicide attempts, depression, and quality of life compared to participants without a history of ACEs.

## Materials and Methods

The original RCT evaluated the efficacy of a new cognitive-behavioral short-term program for adolescent NSSI, the Cutting-Down Programme [CDP (43)], compared to a high-quality treatment as usual (TAU). The detailed protocol was registered at the German Clinical Trials Register (DRKS00003605; http://www.drks.de). In addition, study protocol (44) and the results of the original study (32) have been published elsewhere. The present study investigated the impact of ACEs on treatment outcome within this RCT. To test the mentioned hypotheses, a quasi-experimental study with a between-subject design with repeated measures was conducted.

### Participants and Procedure

The study comprised a sample of 74 participants (mean age 14.9 years, SD = 1.2; 96.0% female) which were recruited through in- and outpatient units at the Clinic of Child and Adolescent Psychiatry at the University Hospital Heidelberg, Germany. Ethical approval was obtained from the institutional review board of the medical faculty at the University of Heidelberg (Ethics Committee No.: S-363/2011). The data analyzed were collected between February 2012 and 2017. Eligible participants were between 12 and 17 years old and were required to have engaged in NSSI on at least 5 days during the past 6 months (DSM-5 criterion A). The last incident of NSSI must not have dated back longer than 1 month. Exclusion criteria were as follows: acute psychotic symptoms; acute intent to harm self or others, which required an intensive psychiatric intervention; an impaired intellectual functioning; receiving current psychotherapeutic treatment. Subjects were included into the study only if both adolescents and caregivers had given their written consent. Before, they were informed about the purposes, content as well as risks, and benefits of the study by an information sheet.

Within the original study, participants were randomly assigned to receive on average 10 sessions of CDP or 19 sessions of treatment as usual (TAU). The CDP was delivered according to the manual by therapists in our specialized outpatient clinic (AtR!Sk), whereas TAU was standard care within the existing mental health care system requiring that TAU therapists agree to provide a first appointment and subsequent therapy within two to 4 weeks. TAU was either cognitive–behavioral therapy or depth psychology. Participants within both groups were able to receive general psychosocial management as well as pharmacological treatment, as needed. All study therapists received training in the CDP beforehand. Within the present study, participants were separated in two groups: participants with at least one ACE and participants with no history of ACEs.

Study participants were assessed at multiple time points: before treatment (T0) and four (T1) and 10 months (T2) after the beginning of the treatment. Participants received monetary compensation for participating in each assessment.

### Assessment Measures

#### Assessment of ACEs

ACEs were assessed at T0 using the Childhood Experience of Care and Abuse (CECA) Interview (45), which is considered to be the gold standard criterion in this field of research. It is a semi-structured interview with an investigator-based approach to rating. Instead of the subject's feelings, behavioral indicators of perpetrators' actions are assessed. The core domains are as follows: parental antipathy, parental neglect, physical abuse, sexual abuse, and psychological abuse. The CECA Interview is a reliable measure both in adults and in adolescents. CECA interviewers receive extensive training before being allowed to use the instrument. Inter-rater reliability was satisfactory both in the English and in the German version (original version: κs = 0.62–1.00; German version: 0.68–1.00) (45, 46).

#### Assessor's Training

In the context of the present study, the clinical psychologist, who conducted the CECA interview, was intensively trained in assessing the interview beforehand.

Training consisted of different aspects:

1. Training manual: there was a training manual with detailed instructions and guidelines about the conduct of the CECA interview including many examples for practice.
2. Training: the clinical psychologist who assessed the CECA interviews was trained by Antonia Bifulco, who developed the CECA, within a two-day workshop comprising practical exercises and ratings.

#### Inter-rater Reliability

To check for inter-rater reliability, 20 (27.0%) audiotaped CECA interviews of the clinician assessing the CECA were assessed by an independent second rater blind for the first rater's scores. Inter-rater reliability was very good (κ = 0.84 for psychological abuse, κ = 0.89 for role reversal, κ = 0.89 for paternal antipathy, and κ = 1.00 for maternal antipathy and neglect, paternal neglect as well as physical and sexual abuse).

#### Outcome Measures

NSSI and suicide attempts were assessed with the German version of the Self-Injurious Thoughts and Behaviors Interview (SITBI-G) (47, 48) at T0, T1, and T2. Common comorbid mental disorders were assessed at T0 using the German version of the Mini-International Neuropsychiatric Interview for children and adolescents (M.I.N.I.-KID 6.0) (49) and parts of the Structured Clinical Interview for DSM-IV-Axis II (SKID-II) (50). Criteria of the following personality disorders were assessed: avoidant, dependent, borderline, and antisocial personality disorder.

In addition, the following self-report measures were used for study assessment at T0, T1, and T2: participants reported on depression symptoms using the German version of the Beck Depression Inventory II (BDI-II) (51). To assess subjective health and well-being at all three evaluations, participants filled out the KIDSCREEN-27 questionnaire (52). For further information on the assessment measures, see the detailed and published original study (32).

### Statistical Analysis

Descriptive analyses were used to characterize the baseline study sample. Nominal data are presented as frequencies, while continuous data are presented as mean and standard deviation (SD). For variables with highly askew distribution, data are presented as medians and interquartile ranges.

The changes in NSSI over time were analyzed with mixed-effect negative binomial regression because of the overdispersion of rates. Changes in depressive symptoms, suicide attempts, and quality of life over time were analyzed with mixed-effect multilevel regression.

A mixed-effect negative binomial regression was calculated to investigate the impact of single forms of ACE on NSSI. Subsequently, a stepwise regression model was conducted in order to minimize the Bayes Information Criterion (BIC). Thus, single ACE forms with lower independent effects on NSSI were gradually taken out of the model. Pearson correlations were calculated to describe the inter-correlations of ACEs (see Supplementary Material). The analyses were performed with Stata (version 15; StataCorp LLC, College Station, TX, USA).

## Results

### Prevalence of ACEs and Sociodemographic Characteristics

Based on the CECA interview, participants were separated in two groups: 30 patients (40.5%) reported at least one ACE. This compared with 44 participants (59.5%) with no history of ACEs (*p* = 0.108).

Antipathy was the most common form of ACEs (*n* = 28, 93.3%). Maternal antipathy (*n* = 17, 56.7%) was more common than paternal antipathy (*n* = 11, 36.7%) within the ACE group. The second leading form of ACEs was neglect (*n* = 16, 53.3%). Here, paternal neglect was more common (*n* = 11, 36.7%) than maternal neglect (*n* = 5, 16.7%). Detailed information on all ACE frequencies as well as baseline demographic and clinical characteristics regarding the two groups is shown in Table 1. There were no differences in the baseline demographic characteristics and diagnostic variables between the two groups. Table 2 includes all outcome variables at different time points. There was a marginal significant difference concerning NSSI within the past 6 months at T2 between the ACE and no-ACE group. The ACE group showed a marginal significant greater reduction in NSSI frequency X. Concerning depression and quality of life, there were no differences between the groups. Importantly, there was no difference between the ACE and no-ACE group concerning the use of interventions (number of sessions completed; *p* = 0.236).

**Table 1:** Sociodemographic and clinical characteristics of participants by ACEs at T0.

**Sociodemographic variable/diagnostic category**	**No ACE (*****N*** **= 44)**		**ACE (*****N*** **= 30)**		**Total (*****N*** **= 74)**		**Group differences**
**Age**	**M**	**SD^a^**	**M**	**SD**	**M**	**SD**	***p*-value^d^**
	14.7	1.2	15.2	1.2	14.9	1.2	0.108
**Sex**	***N***	**%**	***N***	**%**	***N***	**%**	***p*****-value**
Female	41	93.2	30	100.0	71	96.0	0.144
Male	3	6.8	0	0.0	3	4.1	
**School type^b^**	***N***	**%**	***N***	**%**	***N***	**%**	***p*****-value**
Gymnasium	21	47.7	12	40.0	33	44.6	0.708
Realschule	16	36.4	14	46.7	30	40.5	
Förderschule/Hauptschule	7	15.9	4	13.3	11	14.9	
**ACEs**	**N**	**%**	**N**	**%**	**N**	**%**	
Antipathy mother/mother figure	**–**		17	56.7	17	56.7	
Antipathy father/father figure	**–**		11	36.7	11	36.7	
Neglect mother/mother figure	**–**		5	16.7	5	16.7	
Neglect father/father figure	**–**		11	36.7	11	36.7	
Physical abuse mother/ mother figure	**–**		6	20.0	6	20.0	
Physical abuse father/father figure	**–**		4	13.3	4	13.3	
Physical abuse both Parents	**–**		3	10.0	3	10.0	
Sexual abuse	**–**		4	13.3	4	13.3	
Psychological abuse	**–**		5	16.7	5	16.7	
Role reversal	**–**		7	23.3	7	23.3	
**M.I.N.I.-Kid Primary Diagnoses^c^**	***N***	**%**	***N***	**%**	***N***	**%**	***p*****-value**
No diagnosis	3	6.8	0	0.0	3	4.1	0.265
Current major depression	16	36.4	11	36.7	27	36.5	
Past major depression	1	2.3	2	6.7	3	4.1	
Recurrent depressive disorder	4	9.1	4	13.3	8	10.8	
Dysthymia	12	27.3	4	13.3	16	21.6	
Agoraphobia	1	2.3	0	0.0	1	1.4	
Social phobias	2	4.6	0	0.0	2	2.7	
Post traumatic stress disorder	0	0.0	2	6.7	2	2.7	
Drug/alcohol dependence	0	0.0	1	3.3	1	1.4	
ADHD	0	0.0	1	3.3	1	1.4	
Oppositional defiant disorder	1	2.3	2	6.7	3	4.1	
Affective disorders with psychotic features	1	2.3	0	0.0	1	1.4	
Bulimia nervosa	0	0.0	1	3.3	1	1.4	
Adjustment disorders	3	6.8	2	6.7	5	6.8	
**SKID-II**							
Borderline personality disorder	10	22.7	13	43.3	23	31.1	0.061

a *SD, standard deviation*.

b *Foerderschule, school for students with special needs; Hauptschule, 9 years of elementary school; Realschule, 6 years of school after 4 years of elementary school, terminating with a secondary school level-I certificate; Gymnasium, 8 years of school after 4 years of elementary school, terminating with the general Qualification For University entrance*.

c *Multiple diagnoses per subject possible*.

d *Baseline group differences regarding sociodemographic variables and diagnostic categories*.

**Table 2:** Treatment and clinical outcomes by ACEs.

**Intervention/clinical outcome**	**No ACE**		**ACE**		**Group differences**
**NSSI in last 6 months**	**Median**	**IQR^a^**	**Median**	**IQR**	***p*-value**
T0	60	30–90	50	20–120	0.054
T1	40	15–100	30	11–72.5	
T2	13.5	1.5–56	4	2–13	
**BDI–II scores**	***M***	**SD**	***M***	**SD**	***p*****-value**
T0	32.0	11.2	34.0	10.6	0.996
T1	27.0	12.8	24.5	15.4	
T2	21.9	14.9	21.8	13.9	
**KIDSCREEN-27**	***M***	**SD**	***M***	**SD**	***p*****-value**
T0	39.4	6.2	37.3	6.1	0.489
T1	41.7	6.6	42.1	7.6	
T2	44.5	7.9	43.8	9.5	

a *Interquartile range*.

### Adverse Childhood Experiences and NSSI

**Figure 1:**
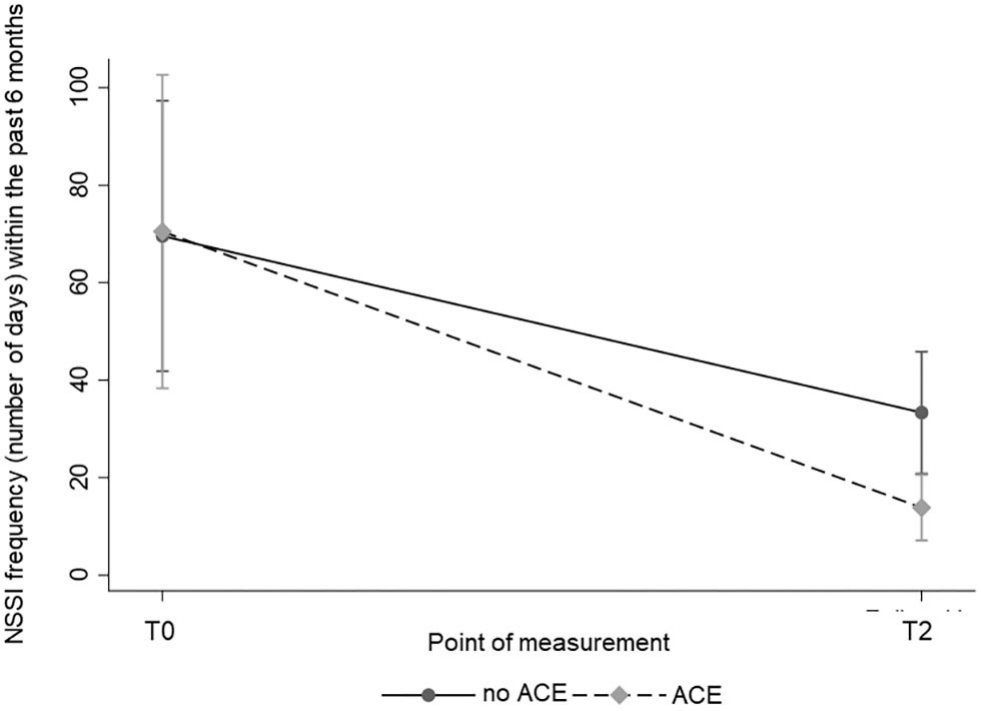
Course of NSSI frequencies (number of days) within the past 6 months by ACE over time.

Regarding NSSI frequencies, both the participants with ACEs and participants without ACEs reached a significant reduction within the past 6 months over time [χ^2^_(1)_ = 26.72; *p* < 0.001] with a marginal significant difference between the two groups in favor of the ACE group [χ^2^_(1)_ = 3.70; *p* = 0.054; Table 2]. A significant point of measurement × ACE interaction [χ^2^_(1)_ = 5.08; *p* = 0.024] regarding the frequency of NSSI within the past 6 months was found. Thus, participants with ACEs reached a greater reduction in the frequency of NSSI than participants without ACEs. The course of NSSI frequency is shown in Figure 1. We also investigated the impact of therapy group affiliation, since the participants received either a specific short-term therapy on NSSI or treatment as usual (32). No interaction with treatment group affiliation was found, indicating that the treatment received did not affect our results. Furthermore, we reanalyzed the data controlling for depression and BPD, which did not change the results. Thus, results without covariates are presented.

To additionally investigate the impact of different types of ACEs on the course of NSSI, we performed a mixed-effect negative binomial regression. Only one form of ACEs, namely, paternal neglect, reached significance for reduction of NSSI frequency within the model [χ^2^_(1)_ = 13.21; *p* < 0.001]. This variable also showed a significant point of time × paternal neglect interaction [χ^2^_(1)_ = 4.50; *p* = 0.034]. However, performing a stepwise regression, no single form of ACEs remained within the model, suggesting that there is no specific type of ACE that was responsible for the overall effect in this study.

### Adverse Childhood Experiences and Suicide Attempts

A significant reduction of suicide attempts could be reached by both groups [χ^2^_(2)_ = 12.67; *p* = 0.002]. Again, there was no difference between the ACE and no-ACE group [χ^2^_(1)_ = 3.21; *p* = 0.073] and no significant point of measurement × ACE interaction [χ^2^_(1)_ = 2.95; *p* = 0.086].

### Adverse Childhood Experiences and Depression

Both participants with or without ACEs reached a significant reduction concerning depressive symptoms [χ^2^_(2)_ = 56.46; *p* < 0.001] without any difference between the two groups [χ^2^_(1)_ = 0.00; *p* = 0.996; Table 2] and no significant point of measurement × ACE interaction [χ^2^_(2)_ = 1.81; *p* = 0.404].

### Adverse Childhood Experiences and Quality of Life

Concerning quality of life, both groups were able to improve this aspect significantly [χ^2^_(2)_ = 44.62; *p* < 0.001]; however, there was no difference between the two groups [χ^2^_(1)_ = 0.24; *p* = 0.628; Table 2]. Again, no significant point of measurement × ACE interaction was found [χ^2^_(2)_ = 2.23; *p* = 0.328].

## Discussion

The purpose of this secondary analysis was to examine the impact of ACEs on therapy outcome within an RCT on adolescent NSSI. According to research, ACEs are a common risk factor for NSSI (10, 53–55). In the present study, 40.5% (*n* = 30) of participants reported a history of ACEs. This prevalence was somewhat smaller than those found in previous studies [64.0% (7), 79% (56)]. However, other studies assessed ACEs using questionnaires while the CECA interview was applied in the present study (45, 46), which thoroughly assesses behavioral indicators of care and abuse rather than not only the subject's feelings. This standardized and strict procedure might explain the lower prevalence of ACEs in the present study.

The most common forms of ACEs were antipathy, in particular maternal antipathy, and neglect, especially paternal neglect. These findings are in line with previous studies showing that antipathy, neglect, parental critique, apathy, and emotional abuse were highly associated with NSSI (7, 18, 19, 22, 23).

Contrary to our hypothesis and contrary to former research (38–41), there was a significant, positive association between ACEs and treatment outcome concerning NSSI frequency. Thus, ACEs were not an unfavorable factor concerning treatment outcome. In fact, the opposite finding emerged. Participants with a history of ACEs showed a greater reduction in NSSI frequency compared to participants without a history of ACEs. Furthermore, both groups reached a significant improvement in suicide attempts, depression, and quality of life with no differences between the two groups. Thus, the ACE group was not inferior to participants without a history of ACEs concerning any other treatment outcomes.

According to the etiology model of Nock (57), which considers the interaction between adverse environmental factors and genetic predisposition, it can be assumed that there might be a stronger impact of biological aspects on NSSI patients without a history of ACEs compared to those with a history of ACEs. Thus, the impact of psychotherapy on the biological vulnerability might be smaller than its impact on environmental factors. These considerations are in line with findings from Nemeroff et al. (58). Traumatized patients with depressive symptoms responded significantly better to a combination of cognitive behavior therapy and pharmacotherapy than to pharmacotherapy alone.

The focus of every treatment applied in the present study (CDP, CBT, etc.) is to learn emotion regulation strategies. These strategies have been reported to be underdeveloped in patients with ACEs compared to patients without ACEs (54). In accordance to these findings, Kaess et al. (7) showed that some forms of ACEs are associated with automatic functions of NSSI, like emotion regulation (59). Thus, there might be a stronger response to these interventions in patients with a history of ACEs.

Similar surprising findings were reported in another study examining BPD patients (60). Patients with less ACEs and a better mother–child relationship reported more suicide attempts than patients with a history of ACEs and a bad mother–child relationship. It was suggested that patients living under good circumstances showed greater hopelessness compared to their counterparts because of not performing well in spite of good living conditions. Another study investigating adolescents within a community sample found that those reporting NSSI experienced a significant increase in the quality of relationships with their fathers. This finding offers empirical support for the social positive reinforcement function of NSSI and might add information to the surprising findings (61).

Another reason might be heightened therapy motivation in patients with a history of ACEs. Therapy motivation was found to be an important factor for therapy success across different disorders (62, 63). Due to higher psychological strain, participants with ACEs might be more motivated than patients without ACEs. In addition, higher rates of hopelessness in participants without ACEs might decrease therapy motivation in these patients. A recent study in male youth (Mean age: 14.7, SD = 1.5) with ACEs living in a residential home found that adolescents with four or more ACEs showed higher rates of treatment engagement (64). In addition, Steinke and Derrick (64) found that patients with a history of abuse had higher levels of readiness to change at admission than those with no history of abuse. The same might be true in the present sample.

In addition, the concept of differential susceptibility extends the understanding that negative environments and ACEs exert negative effects, such as poor treatment outcomes, on children or adolescents presumed “environmentally vulnerable.” In fact, it reflects that heightened susceptibility to negative effects of ACEs and negative environments may also mean heightened susceptibility to positive and supportive environments (65). Thus, adolescents with a history of ACEs may benefit in particular from caring interactions such as psychotherapeutic interventions. This could explain the faster improvement concerning NSSI in adolescents with ACEs compared to adolescents without ACEs.

With these considerations in mind, it would be helpful to instruct parents to provide more caring interactions, as studies found that perceived family support appears to be an important safeguard against NSSI (66). As a clinical implication, existing treatment approaches should also focus on parents as paternal antipathy and emotional neglect seem to be crucial risk factors for NSSI. To meet this point, our working group started to develop a corresponding manual for parents to enrich the Cutting Down Program. Within DBT-A, participation of parents in group and individual therapy is already a fixed component, which seems to be relevant following existing findings.

### Limitations

The study has several limitations. First, the limited sample size does not allow us to do meaningful differential analyses on type, severity, or chronicity of ACEs. Further research should focus on this point. Moreover, the sample consisted predominantly of female participants, which did not allow drawing conclusions on possible effects for males or gender differences. However, considering that female gender has been identified as a risk factor for NSSI, the presented sample depicts this finding (3, 67). Concerning the analyses of single ACE forms, it needs to be considered that the types of ACEs were not equally distributed, which might depict reality on the one hand but generated small subgroups which could have contributed to a lack of significant results on the other hand. Thus, also findings on paternal neglect should be interpreted as explorative, especially since no single ACE remained within the model after stepwise regression. Paternal neglect could be investigated in further studies in particular.

A particular strength of the present study is the participation ratio. There was no dropout from research. Furthermore, the CECA interview was used to assess ACEs. The CECA interview is recognized as the gold standard in this field of research with good reliability and validity (68). Many previous studies solely assessed subjects' feelings by using questionnaires. However, it should be taken into account that the potential risk of a recall bias may still have influenced the present findings.

## Conclusion

With these reservations in mind, this study suggests that participants with ACEs showed similar, and in terms of NSSI even greater improvements during psychotherapeutic treatment compared to participants without a history of ACEs. Considering the essential association between ACEs and NSSI, the present findings possess valuable information for practitioners confronted with adolescents engaging in NSSI. In particular, in the context of a personalized medicine, the identification of specific predictors is crucial to increasing treatment effectiveness (33). In this case, adolescents with NSSI and a history of ACEs may be particularly susceptible to psychotherapeutic treatment and do not seem to represent a group of poorer treatment response as initially expected. In contrast, it may rather be those individuals engaging in NSSI despite no history of ACEs (and a potentially higher biological vulnerability) that may require different or additional treatment options. While further exploration of this relationship with larger samples is required, future research should also consider the impact of single forms of ACEs on treatment outcome.

## Data Availability Statement

The raw data supporting the conclusions of this article will be made available by the authors, without undue reservation.

## Ethics Statement

The studies involving human participants were reviewed and approved by the institutional review board of the medical faculty at the University of Heidelberg (Ethics Committee No.: S-363/2011). Written informed consent to participate in this study was provided by the participants' legal guardian/next of kin.

## Author Contributions

AE wrote the first draft. GF-W participated in recruitment and assessment. PP participated in the design of the study and performed the statistical analyses. RB and FR participated in the design of the study and supervised the study procedure. MK was responsible for the study design and coordination. All authors revised the article critically and approved the final version of the manuscript.

## Conflict of Interest

The authors declare that the research was conducted in the absence of any commercial or financial relationships that could be construed as a potential conflict of interest.
