# Supplementary material for: The Impact of Adverse Childhood Experiences on Therapy Outcome in Adolescents Engaging in Nonsuicidal Self-Injury
Source: Front Psychiatry. 2020 Nov 4;11:505661. doi: 10.3389/fpsyt.2020.505661 (PMC7672012; doi:10.3389/fpsyt.2020.505661)
Supplement: Supplementary file 1 [file Table_1.DOCX]

Supplementary Material

Pearson’s correlations and respective p-values for inter-correlations of ACEs

| **ACEs** | **MA** | **PA** | **MN** | **PN** | **PhA** | **SA** | **PsA** | **RR** |
| --- | --- | --- | --- | --- | --- | --- | --- | --- |
| **MA** | 1.00 |  |  |  |  |  |  |  |
| **PA** | .30* | 1.00 |  |  |  |  |  |  |
| **MN** | .37* | .04 | 1.00 |  |  |  |  |  |
| **PN** | .11 | .34* | .19* | 1.00 |  |  |  |  |
| **PhA** | .51* | .32* | .31* | .11 | 1.00 |  |  |  |
| **SA** | .02 | .24* | .17* | .24* | .37* | 1.00 |  |  |
| **PsA** | .24* | .35* | .36* | .19* | .59* | .41* | 1.00 |  |
| **RR** | .36* | .13* | .67* | .27* | .57* | .34* | .67* | 1.00 |

MA: maternal antipathy, PA: paternal antipathy, MN: maternal neglect, PN: paternal neglect, PhA: physical abuse, SA: sexual abuse, PsA: psychological abuse, RR: role reversal

* *p* < .05
